# Paracrine Senescence of Mesenchymal Stromal Cells Involves Inflammatory Cytokines and the NF-κB Pathway

**DOI:** 10.3390/cells11203324

**Published:** 2022-10-21

**Authors:** Lun-Yin Chou, Chun-Te Ho, Shih-Chieh Hung

**Affiliations:** 1 Integrative Stem Cell Center, China Medical University Hospital, Taichung 404, Taiwan; 2 Drug Development Center, Institute of Translational Medicine and New Drug Development, School of Medicine, China Medical University, Taichung 406, Taiwan; 3 Department of Orthopaedics, China Medical University Hospital, Taichung 404, Taiwan

**Keywords:** mesenchymal stromal cells, paracrine, IL-1α, IL-8, NF-κB, senescence, senescence-associated secretory phenotype (SASP)

## Abstract

It has been known that senescence-associated secretory phenotype (SASP) triggers senescence of the surrounding normal cells. However, SASP signaling regarding mesenchymal stromal cell aging remains to be fully elucidated. Therefore, the present study aimed to clarify the molecular mechanism of late (passage) MSC-induced paracrine SASP-mediated senescence of early (passage) MSCs during ex vivo expansion. Here, we conducted an extensive characterization of senescence features in bone-marrow (BM)-derived MSCs from healthy human donors. Late MSCs displayed an enlarged senescent-like morphology, induced SASP-related proinflammatory cytokines (IL-1α and IL-8), and reduced clonogenic capacity and osteogenic differentiation when compared to early MSCs. Of note, paracrine effects of SASP-related IL-1α and IL-8 from late MSCs induced cellular senescence of early MSCs via an NF-κB-dependent manner. Moreover, cellular senescence of early MSCs was promoted by the synergistic action of IL-1α and IL-8. However, inhibition of NF-κB by shRNA transfection or using inhibitors in early MSCs blocked early MSCs cellular senescence caused by paracrine SASP of late MSCs. In conclusion, these findings reveal that late MSCs display features of senescence and that, during ex vivo expansion, SASP-related proinflammatory cytokines contribute to activate a cellular senescence program in early MSCs that may ultimately impair their functionality.

## 1. Introduction

Since the first clinical trial of human mesenchymal stromal cells (hMSCs)-based therapy in 1995, hMSCs have become the most clinically studied cell therapy candidates. MSCs are capable of self-renewal and have been shown to mediate potent immunomodulatory effects to influence both innate and adaptive immune cells. The unique immunomodulatory properties of MSCs have expanded their therapeutic capacity [1]. However, MSC senescence and pro-inflammatory activation are involved in the loss of therapeutic potential toward a consequent decline in quality [2]. Although efforts have been made to identify putative markers for predicting senescence of mesenchymal progenitor cells upon expansion, methods to remove senescent cells prior to application need to be developed.

Notably, aged MSCs (isolated from aged donor) comprise a heterogeneous population, with a high proportion of senescent cells compared with cells isolated from young donors (defined as young MSCs), and concomitant with reduced proliferation and differentiation capacity [3,4], which characterizes MSCs from donors of different ages and age-associated changes in MSC marker profiles. Aged MSCs display increased senescent features such as enhanced activity of senescence-associated-beta-galactosidase (SA-β-gal), oxidative damage and over-expression of p21, p16 and p53, which may lead to cell cycle arrest, telomere shorting, senescence, and impaired replicative capacity [5,6,7]. Moreover, aged MSCs have dysregulated glucose utilization and mitochondrial respiratory function [8].

As MSCs age, these cells are able to exert their effects in the surrounding environment and modulate their niche as a result of a unique secretome profile, which is termed the “senescence-associated secretory phenotype” or SASP [9,10]. The senescent MSCs spread the SASPs to neighboring cells via the paracrine mechanism, hence accelerating the aging process [11,12]. The increases in SASP factors such as interleukin 1-alpha (IL-1α), IL-6, IL-8, and monocyte chemotactic protein 1 (MCP1) in the conditioned medium of aged MSC culture reduce cell proliferation and differentiation [13]. Additionally, the immunomodulatory capacity of senescent MSCs has been reported to be associated with an increase in IL-6 expression and diminished protective immunosuppressive function [2]. Consequently, neutralizing such pro-inflammatory cytokines has been shown to restore the immunosuppressive function of MSCs isolated from elderly patients with atherosclerosis [14].

Another SASP, nuclear factor-κB (NF-κB), is integrated by several transcription factors implicated in immune and inflammatory responses [15], which are involved in the cellular senescence in embryonic fibroblasts, the TNF-α-induced proinflammatory activation in MSCs [16] and the activation of immune cells during aging [17]. Recently, Liu et al. reported that several central nerve system (CNS) diseases are linked to NF-κB activated by inflammatory mediators. Furthermore, the inhibition of NF-κB has been shown to restore osteogenic differentiation in mouse MSCs treated with TNF-α and IL-17 in vitro [18], indicating that it may be a therapeutic target of MSCs senescence. Similarly, in our previous study, NF-κB was the therapeutic target for endotoxin-induced acute lung injury using MSC-conditioned medium [1]. We also proposed the epigenetic pathway of DNMT1, which modulates the expression of p16 and p21 in MSC senescence [19]. SASP has also been reported to trigger MSCs senescence and activation of p16 and p21. However, a recent study also showed that small cells of umbilical cord blood (UCB)-derived MSCs exhibited delayed cellular senescence by inhibiting Toll-like receptor signaling-mediated SASP activation [20].

The long-term ex vivo expansion of hMSCs results in late passage hMSCs, referred to as late MSCs. Like aged MSCs, late MSCs exhibit increased senescence features, such as over-expression of senescence markers, such as p16, p21, and SA-β-gal, cell cycle arrest, impaired replicative capacity, and reduced osteogenic differentiation potential, compared to early passage hMSCs, referred to as late MSCs [21]. In contrast to aged MSCs with in-creased adipogenic potential, late MSCs had reduced adipogenic potential [21]. Investiga-tion of ex vivo expansion-induced SASP may lead to strategies to modulate lineage com-mitment of MSCs in vivo, which may be useful in the treatment of osteoporosis. Nevertheless, to date, late MSC-induced paracrine SASP-mediated senescence of early MSCs re-mains to be fully elucidated.

In the current study, we demonstrated that paracrine SASPs play pivotal roles in cellular senescence during ex vivo expansion of hMSCs, and clarified the molecular mechanism of late MSCs-induced paracrine SASP-mediated senescence of early MSCs.

## 2. Materials and Methods

### 2.1. Establishment of Bone Marrow-Derived MSC Isolation Procedure

Bone marrow aspirates were collected from three individual donors (Table S1) who received orthopedic surgery at Taipei Veterans General Hospital between 2012 and 2015. The study was approved by the institutional review board (IRB) of Taipei Veterans General Hospital (#2012-05-007A) and followed the guidelines and regulations. The protocols for MSC isolation and expansion were modified from previously described methods [22]. In brief, mononuclear cells were isolated from heparinized bone marrow by density gradient centrifugation using Ficoll-Hypaque (Sigma-Aldrich, St. Louis, MO, USA) with a density of 1.077 g/L, followed by seeding into 6-well plates (Corning Company, Corning, NY, USA) with complete medium (CM: Dulbecco’s Modified Eagle Medium (DMEM; Gibco-BRL, Gaithersburg, MD, USA), supplemented with 10% fetal bovine serum (FBS, Gibco-BRL), 100 units/mL penicillin, 100 μg/mL streptomycin, and 2 mM l-glutamine (Invitrogen, Carlsbad, CA, USA)) at 37 °C under 5% CO_2_ atmosphere. At 9 days after seeding, MSCs were recovered and reseeded in 10 cm plastic dishes at an initial density of 4 × 10^3^ cells/cm^2^. Culture medium was changed twice per week and a sub-culture was performed at 1:3 to 1:5 split every week. Mycoplasma contamination was checked every month. Cells with mycoplasma contamination were discarded.

### 2.2. Senescence-Associated β-Galactosidase (SA-β-gal)

Senescence-associated β-galactosidase (SA-β-gal) staining was performed using a β-galactosidase staining kit (BioVision, Milpitas, CA, USA) according to the manufacturer’s instructions. Briefly, MSCs at passage number 5, 9, and 13 were washed with PBS and fixed with 3.7% formaldehyde for 5 min at room temperature. After washing, the cells were incubated with β-gal staining solution (1 mg/mL, 5-bromo-4-chloro-3-indolyl-b-galactoside) overnight at 37 °C [23]. The SA-β-gal-positive cells were observed in phase contrast images. SA-β-gal-positive cells were observed and acquired at room temperature using an inverted microscope (Nikon Eclipse TE2000, Tokyo, Japan) with a 20X/0.40 objective equipped with a camera (Nikon Corporation, Tokyo, Japan).

### 2.3. Colony Forming Unit (CFU) Assays

The clonogenic ability of MSCs from different passage numbers (early MSCs: passage 2–5 and late MSCs: passage 9–14) was determined by CFU assay. Different numbers of early MSCs were co-cultured with late MSCs. After 14-day incubation, the plastic adherent colonies were then fixed with 10% formalin for 15 min, followed by washing using ddH_2_O for twice and stained with 0.5% crystal violet for 30 min at room temperature. Cell colony counting was performed on a microscope. For quantification of CFU, colonies (whole well) with diameters greater than 1 mm (~40 cells) were counted as previously described [24]. The quantification of crystal violet was performed by solubilizing crystal violet with methanol and measuring the absorbance at 570 nm using a microplate reader (Infinite^®^ M1000 Tecan Ltd., Maennedorf, Switzerland).

### 2.4. Osteogenic Differentiation Potential Assay

For low density differentiation of colonies, early MSCs were plated in a 6-well plate and cultured in CM for 7 days as previously described [25]. For differentiation into osteoblasts, cells were induced in osteogenic induction medium [OIM: CM supplemented with 10^−8^ M dexamethasone, 50 µg/mL ascorbic acid-2 phosphate, 10 mM β-glycerophosphate (Sigma-Aldrich, St. Louis, MO, USA)]. After induction in defined induction medium for 14 days, cells in OIM were stained with Alizarin red staining (ARS, Sigma-Aldrich, St. Louis, MO, USA), followed by extraction and measurement of O.D. values of ARS at 550 nm.

### 2.5. Quantitative Reverse-Transcription PCR (qRT-PCR)

Cells were washed twice with PBS, and total RNA was extracted by PureLink™ RNA Mini Kit according to the manufacturer’s instructions (Invitrogen, Carlsbad, CA, USA). Total RNA was subjected to reverse transcription and then qRT-PCR using SYBR green on CFX96 Optical Reaction Module (Bio-Rad, Hercules, CA, USA). Primer sets used to quantify osteogenic differentiation [26] are listed in Table S2.

### 2.6. Gene Set Enrichment Analysis (GSEA)

Total RNA was isolated with the RNeasy Micro Kit (QIAGEN, Hilden, Germany). Illumina TruSeq RNA sample Prep kit was used to prepare RNA-Seq library. BM-MSCs paired RNA-Seq experiments were from two individuals. More than 30 million (mean ± standard deviation = 37,392,922 ± 3,100,588) 100 bp paired-end reads for each RNA-Seq sample were generated using an Illumina HiSeq 2000 sequencer in National Center for Genome Medicine, Academia Sinica. The human genome assembly hg38 including un-placed and un-localized scaffolds and RefGene annotation were downloaded from the UCSC Genome Browser on 2017.1.248. Sequencing bases with low quality were trimmed based on the Phred + 33 quality score (>20) from both of the 5′- and 3′-ends of reads. After trimming, reads were discarded, if they were shorter than 75 bp, or had one or more ambiguous base. The alignment, quantification, normalization, and differential expression analysis were performed by STAR 2.5.3a49 through Partek Flow (Partek Inc., Chesterfield, MO, USA), htseq-count 0.6.050, TMM51, and edgeR 3.18.152, respectively. Genes with count-per-million (CPM) values above 1 in at least three samples were retained, and genes with no or low expression levels were discarded. The RNAseq data of early and late MSCs were analyzed by GSEA 4.2.1 using the 1615 gene set (https://csibioinfo.nus.edu.sg/csingsportal/login/home.php) (accessed on 17 November 2021). [27,28]. Normalized enrichment scores (NES) and false discovery rates (FDR) were obtained for all variables and signatures. The database for biological processes/pathways, the Reactome pathway database, was used for the analysis. FDR < 0.05 was considered significant. RNA-seq raw data are accessible at NCBI GEO with accession number GSE178804. All relevant data are available from the authors with restrictions.

### 2.7. Enzyme-Linked Immunosorbent Assay (ELISA)

To collect conditioned medium, 8 × 10^5^ MSCs were seeded into plates and cultured until 80% confluence, followed by incubating in serum-free medium for another 48 h [1], followed by filtration using a Millipore Ultra centrifugal unit (UFC 900396, Millipore, Billerica, MA, USA) to concentrate 40 folds. The IL-1α and IL-8 concentrations of MSC conditioned medium was determined using a commercially available ELISA kit (IL-8, BioLegend, San Diego, CA, USA; IL-1α, Abcam, Cambridge, MA, USA) according to the manufacturer’s recommendations. The levels of IL-1α and IL-8 were calculated from the calibrator curves.

### 2.8. Human NF-κB Pathway Array

Early MSCs until reaching 80% confluence were incubated with 10 ng/mL IL-1α or 10 ng/mL IL-8 recombinants (Peprotech, Rocky Hill, NJ, USA), respectively, in serum-free DMEM supplemented with 1% antibiotics for 24 h. The protein expression analysis of early MSCs and pro-inflammatory activation were performed using a Proteome Profiler Human NF-κB Pathway Kit (ARY029, R&D Systems, Minneapolis, MN, USA). All procedures and data analysis followed the manufacturers’ instructions. Briefly, total protein was extracted using Lysis Buffer. Each sample was run on each array (membrane) under incubation at 4 °C overnight on a shaker. The membrane was washed three times with PBS. Detection Antibody Cocktail was added to each membrane followed by 1 h incubation. The membrane was then incubated with Chemiluminescence Reagent Mix and exposed to generate the profile of spot pixel density image. The density signals of each spot were quantified by Image J software (Rasband W; National Institutes of Health, Bethesda, MD, USA). The relative differences of sample signals were normalized with reference spots.

### 2.9. Western Blotting

Cell extracts were prepared with M-PER (Pierce, Rockford, IL, USA) plus protease inhibitor cocktail (Halt™; Pierce, Rockford, IL, USA) and protein concentrations were determined using the BCA assay (Pierce, Rockford, IL, USA) [29]. Aliquots of protein lysates were separated on SDS-8 or 15% polyacrylamide gels and transferred to PVDF membrane filters, which were blocked with 5% blotting-grade milk (Bio-Rad, Hercules, CA, USA) in TBST (20 mM Tris-HCl (pH 7.6), 137 mM NaCl, 0.1% Tween 20) for 1 h. The filters were then incubated 1 h at room temperature with a 1:1000 dilution in TBST of antibodies against p16 (#92803, Cell Signaling Technology, Danvers, MA, USA), p21(sc-6246, Santa Cruz Biotechnology, Santa Cruz, CA, USA), IKK2 (GTX107970, GeneTex, Hsinchu, Taiwan) and NFκB p65 (phospho Ser536) (GTX133899, GeneTex, Taiwan), reacted with corresponding secondary antibodies, and detected using a chemiluminescence assay (Millipore, Billerica, MA, USA).

### 2.10. Lentiviral Vector Production and Cell Infection

The expression plasmids and the bacterial clone for NF-κB p65 shRNA (TRCN0000014683 and TRCN0000014686) were provided by the RNAi core of the National Science Council in Taiwan. Subconfluent cells were infected with lentivirus in the presence of 100 μg/mL protamine sulfate (P4020, Sigma-Aldrich). At 48 h post-infection, puromycin (2 μg/mL) was used to select infected cells for 48 h.

### 2.11. Statistical Analysis

Experimental data were analyzed using GraphPad Prism 8.0 (GraphPad Software, San Diego, CA, USA). Statistical significance was determined using one-way analysis of variance with Tukey’s post hoc multiple comparison test for normally distributed data. Data are expressed as the mean ± SD. Values of *p* < 0.05 were considered statistically significant.

## 3. Results

### 3.1. Paracrine Effects from Late MSCs Suppressed Clonogenic Capacity and Osteogenic Differentiation of Early MSCs

**Figure 1:**
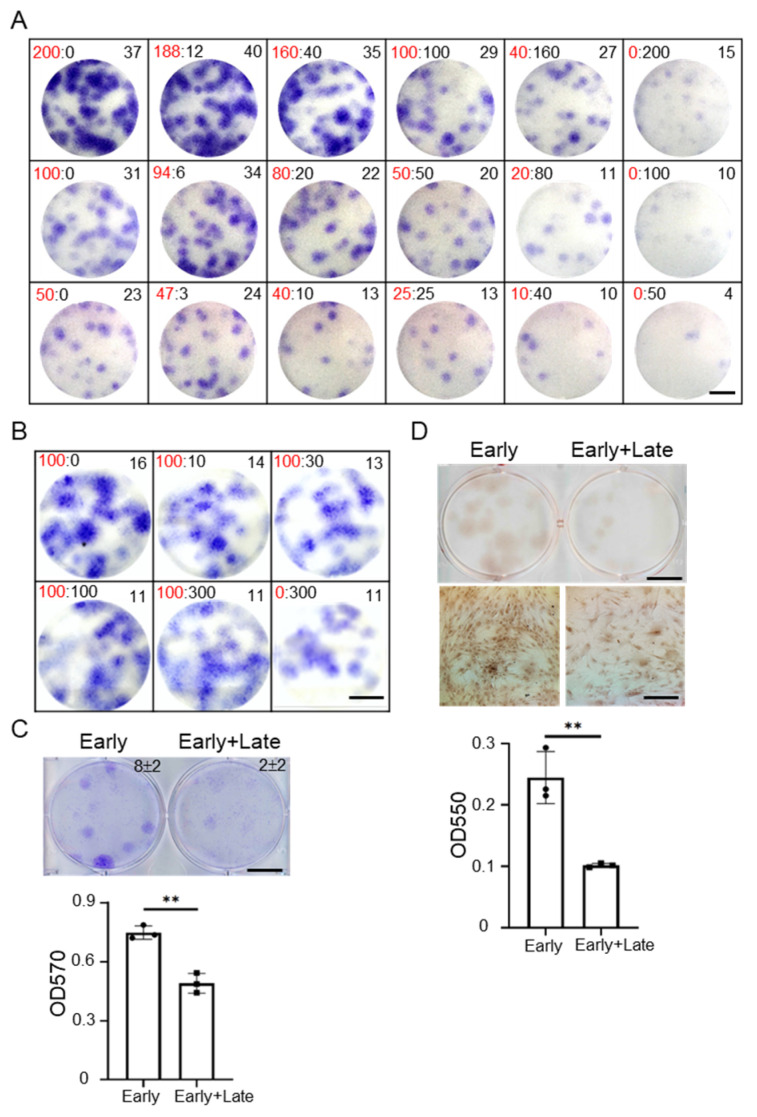
Late MSCs impede proliferation and osteogenic differentiation of early MSCs. (**A**) Early and late MSCs were seeded in each well of 6-well plates at indicated cell numbers (early:late, in left upper site) and cultured for 14 days. Cells were then stained with crystal violet and the colony number for each well is shown in right upper site. Representative data for three independent experiments are shown. (**B**) A total of 100 early MSCs were cultured in the absence or presence of indicated number of late MSCs in 6-well plates and cultured for 14 days. The cells were stained with crystal violet. (**C**) A total of 100 early MSCs were cultured in the absence or presence of 100 late MSCs in 6-well plates and cultured for 14 days. The cells were stained with crystal violet (upper) and the colony number for each well is shown in right upper site. The dye was extracted for OD measurement at 570 nm (lower). Bar = 10 mm. (**D**) A total of 100 early MSCs were cultured in the absence or presence of 100 late MSCs in 6-well plates and cultured for 7 days, and replaced with osteogenic induction media for an additional 14 days. The cells were stained with Alizarin Red-S (upper) and the dye was extracted for OD measurement at 550 nm (lower). Bar = 10 mm (upper); 200 μm (lower). Data are expressed as mean ± SD. (*n* = 3). Asterisks indicate significant differences as determined by the unpaired Student’s *t*-test (** *p* < 0.01 versus early MSCs).

**Figure 2:**
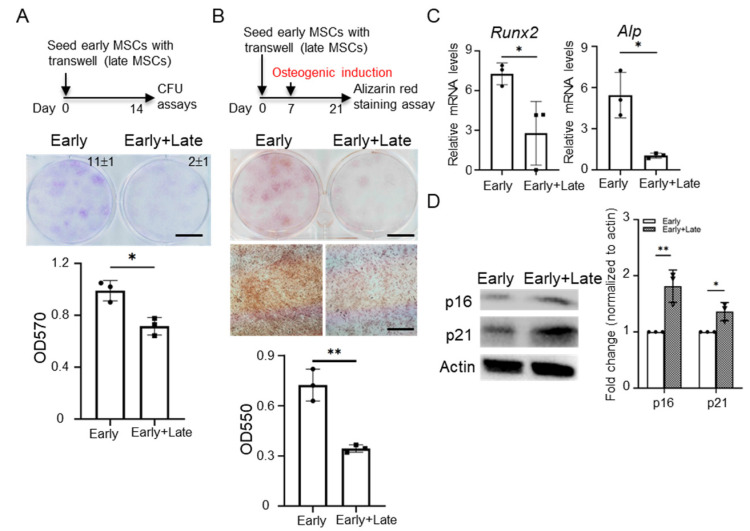
Late MSCs impede proliferation and osteogenic differentiation of early MSCs through paracrine effects. (**A**) A total of 100 early MSCs were seeded in each lower well of 6-well plates in the absence or presence of 100 late MSCs seeded in the upper well of Transwell (0.4 μm pore size insert) and cultured for 14 days. The cells were stained with crystal violet (upper) and the colony number for each well is shown in right upper site. The dye was extracted for OD measurement at 570 nm (lower). Bar = 10 mm. (**B**) A total of 100 early MSCs were seeded in each lower well of 6-well plates in the absence or presence of 100 late MSCs seeded in the upper well of Transwell and cultured for 7 days, and replaced with osteogenic induction media for an additional 14 days. The cells were stained with Alizarin Red-S (upper) and the dye was extracted for OD measurement at 550 nm (lower). Bar = 10 mm (upper); 200 μm (lower). (**C**) A total of 5 × 10^4^ early MSCs were cultured in each lower well of 6-well plates in the absence or presence of 5 × 10^4^ late MSCs seeded in the upper well of Transwell and cultured in osteogenic induction media for 3 days. qPCR analysis of transcript levels for genes associated with osteogenesis. Transcript levels were normalized based on GAPDH amplification. Alkaline-p (Alp). *n* = 3 independent experiments. (**D**) A total of 5 × 10^4^ early MSCs were cultured in each lower well of 6-well plates in the absence or presence of 5 × 10^4^ late MSCs seeded in the upper well of Transwell and cultured in growth media for 3 days. The senescence markers, p16 and p21, of early MSCs were analyzed by Western blotting. Actin served as the protein loading control. Fold change to Actin with normalization is shown. Data are expressed as mean ± SD (*n* = 3). Asterisks indicate significant differences as determined by the unpaired Student’s *t*-test (* *p* < 0.05, ** *p* < 0.01 versus early MSCs).

Many studies indicate that accumulating senescent MSCs in pathogenetic tissue increases senescent features such as enhanced SA-β-gal activity and high level of p21 and p16 [5,6,7]. We hypothesized that late MSCs may affect senescence, proliferation and differentiation capacities of early MSCs. For this purpose, we first compared the senescent features between MSCs at early (P2–5) and late (P9–14) passages. Late MSCs were characterized by a large and flat morphology, increased expression of senescence markers (SA-β-gal activity, p16 and p21), and cell cycle arrest (Figure S1), indicating that they have undergone senescence [30]. At co-cultivation with fixed total cell numbers, the increase in the number of late MSCs reduced the clonogenic capacity of MSCs (Figure 1A). In addition, at co-cultivation of late MSCs and fixed cell numbers of early MSCs, the increase in the number of late MSCs also impaired the clonogenic capacity of MSCs (Figure 1B). Similarly, more reduced clonogenic capacity (early MSCs: 8 ± 2 > late MSCs: 2 ± 2, *p* value: 0.0175) and impaired osteogenic capacity were also observed in co-cultivation with late MSCs and early MSCs compared with that in early MSCs alone (Figure 1C,D). A Transwell co-culture system with 0.4 μm pore size was used to investigate paracrine action. First, 100 early MSCs were seeded in each lower well of 6-well plates in the absence or presence of 100 late MSCs seeded in the upper well of Transwell and cultured for 14 days. Similarly, the paracrine effects of late MSCs suppressed the clonogenic capacity (early MSCs: 11 ± 1 > late MSCs: 2 ± 1, *p* value: 0.0002, Figure 2A), osteogenic gene expression (*Runx2 and Alp*) and osteogenic capacity of early MSCs (Figure 2B,C), and increased senescence markers (Figure 2D). These data indicate that paracrine molecules of late MSCs inhibit the stemness property of early MSCs.

### 3.2. Late MSCs Induce the Expression of SASP-Related Proinflammatory Molecules

**Figure 3:**
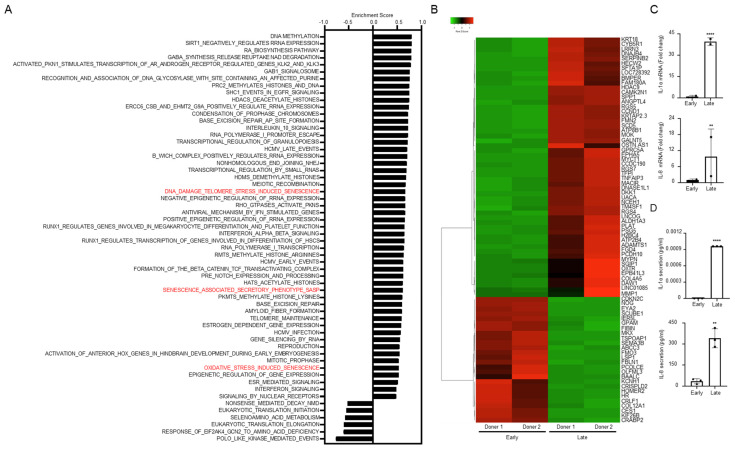
Gene set enrichment analysis (GSEA) reveals several pathways upregulated or downregulated in late MSCs. Early or late MSCs from two individual donors were subjected to: (**A**) RNA-seq for gene expression profile analysis and subsequent Gene Set Enrichment Analysis (GSEA) using Reactome pathways for a gene score file at FDR < 0.05. Positive and negative scores indicate upregulated and downregulated pathways in late MSCs, respectively. (**B**) The top 80 genes with FDR < 0.05 are shown in RNA-seq heatmap. (**C**) The mRNA expression of IL-1α and IL-8 in early or late MSCs from two individual donors was shown, respectively (*n* = 2). (**D**) 8 × 10^5^ early MSCs or late MSCs were grown in serum-free condition in each 10 cm dish for 72 h, the conditioned media were harvested for the detection of IL-1α and IL-8 secretion by enzyme-linked immunosorbent assay (ELISA). Data are expressed as mean ± SD (*n* = 3). Asterisks indicate significant differences as determined by the unpaired Student’s *t*-test (** *p* < 0.01, **** *p* < 0.0001 versus early MSCs).

To identify gene expression patterns connected with MSC aging, the RNAseq data of the late cells (donor 1: passage 9; donor 2: passage 14) and Gene Set Enrichment Analysis (GSEA) were performed. GSEA revealed that multiple pro-inflammatory pathways were activated in late MSCs in comparison to early MSCs (donor 1: passage 2; donor 2: passage 2). The senescence-related gene sets including DNA damage telomere stress-induced senescence, senescence-associated secretory phenotype (SASP) and oxidative stress-induced senescence pathway were significantly increased (FDR < 0.05, Figure 3A). The top 80 genes with FDR < 0.05 from total gene sets were shown in RNA-seq heatmap (Figure 3B). Notably, SASP pathway revealed upregulated pro-inflammatory cytokines, IL-1α (FDR < 0.001) and IL-8, also known as CXCL8 (FDR = 0.011) (Table 1), and further confirmed the levels of conditioned media by q-RT-PCR and ELISA assay (Figure 3C,D). These results suggest that the pro-inflammatory phenotype is acquired in late MSCs compared to early MSCs.

### 3.3. SASP-Related Proinflammatory Cytokines Prompt Senescence in MSCs

**Figure 4:**
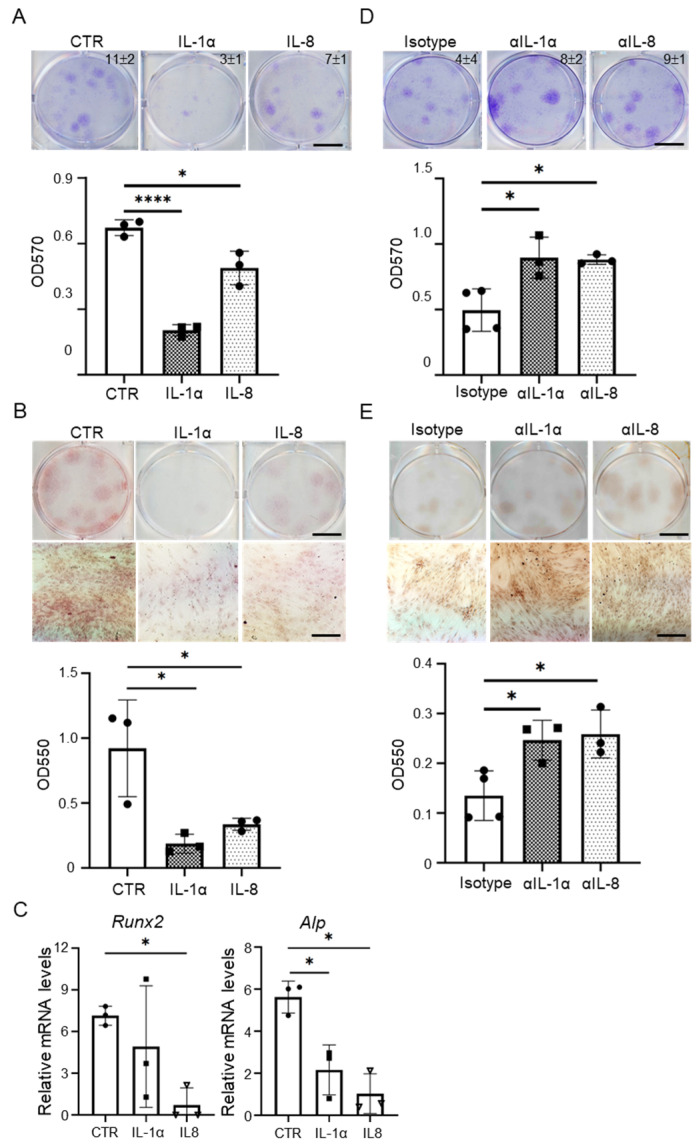
IL-1α or IL-8 induces senescence in MSCs. (**A**) A total of 100 early MSCs were cultured in each well of 6-well plates in the absence or presence of IL-1α (10 ng/mL) or IL-8 (10 ng/mL) and cultured for 14 days. The cells were stained with crystal violet (upper) and the colony number for each well is shown in right upper site. The dye was extracted for OD measurement at 570 nm (lower). Bar = 10 mm. (**B**) A total of 100 early MSCs were cultured in the absence or presence of IL-1α or IL-8 in 6-well plates, the cells were cultured in growth medium for 7 days, and replaced with osteogenic induction media for an additional 14 days. The cells were stained with Alizarin Red-S (upper) and the dye was extracted for OD measurement at 550 nm (lower). Data are expressed as mean ± SD (*n* = 3). Bar = 10 mm (upper); 200 μm (lower). Asterisks indicate significant differences as determined by One Way ANOVA (* *p* < 0.05 and **** *p* < 0.0001 versus control group, CTR). (**C**) A total of 5 × 10^4^ early MSCs were cultured in osteogenic induction media without or with IL-1α or IL-8 in 6-well plates for 3 days. qPCR analysis of transcript levels for genes associated with osteogenesis. Transcript levels were normalized based on GAPDH amplification. Alkaline-p (Alp). *n* = 3 independent experiments. (**D**) A total of 100 early MSCs were cultured in the presence of 100 late MSCs in each well of 6-well plates and cultured with isotype, anti-IL1α or anti-IL-8 antibodies (30 ng/mL) for 14 days. The cells were stained with crystal violet (upper) and the colony number for each well is shown in right upper site. The dye was extracted for OD measurement at 570 nm (lower). Bar = 10 mm. (**E**) A total of 100 early MSCs were cultured in the presence of 100 late MSCs in each well of 6-well plates and cultured with isotype, IL1α or IL-8 antibodies (30 ng/mL, respectively) for 7 days, and replaced with osteogenic induction media for additional 14 days. The cells were stained with Alizarin Red-S (upper) and the dye was extracted for OD measurement at 550 nm (lower). Bar = 10 mm (upper); 200 μm (lower). Data are expressed as mean ± SD (*n* = 3). Asterisks indicate significant differences as determined by One Way ANOVA (* *p* < 0.05 versus isotype group).

The SASP includes higher secretion of pro-inflammatory cytokines, such as IL-1α and IL-8 [31]. When the effects of IL-1α and IL-8 in the properties of MSCs were further assessed, the levels of SA-β-gal were increased in early MSCs treated with IL-1α or IL-8 compared with that in untreated early MSCs, as shown in Figure S2. Subsequent, IL-1α or IL-8 suppressed the clonogenic capacity (Control: 11 ± 2 > IL-1α: 3 ± 1 and IL-8: 7 ± 1, *p* value: 0.0002 and 0.0105, Figure 4A). Then, osteogenic gene expression and osteogenic differentiation were repressed in early MSCs treated with IL-1α or IL-8 compared with that in untreated early MSCs (all *p* < 0.05, Figure 4B,C). In the presence of late MSCs, the clonogenic capacity (Isotype: 4 ± 4 < IL-1α antibody: 8 ± 2 and IL-8 antibody: 9 ± 1) and osteogenic differentiation were induced in early MSCs neutralized with IL-1α or IL-8 antibodies compared with that in early MSCs treated with isotype control (all *p* < 0.05, Figure 4D,E). These data suggest that IL-1α/IL-1R or IL-8/IL-8R signaling pathway may be involved in the increase in senescence features in MSCs.

### 3.4. SASP-Related Proinflammatory Molecules Promote Clonogenic Capacity and Osteogenic Differentiation of Early MSCs via NF-κB Signaling

**Figure 5:**
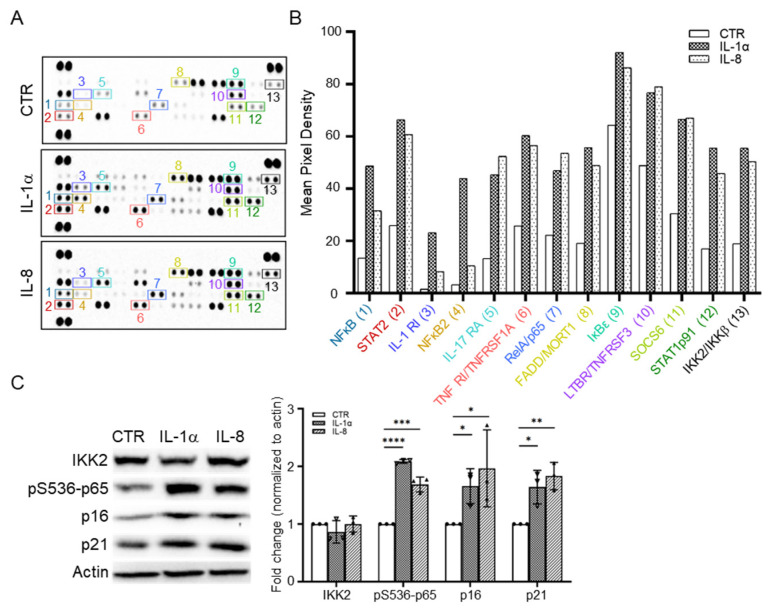
IL-1α or IL-8 upregulates the NFκB signaling pathway in early MSCs. Early MSCs at 50% confluence were treated with IL-1α (10 ng/mL) or IL-8 (10 ng/mL) in serum-free medium in a 10 cm dish for 24 h, followed by (**A**) measurement of protein level by proteome profiler human NFκB pathway array kit and (**B**) quantification of mean pixel intensity by Image J software. The labeled spots are: 1. NFκB1, 2. STAT2, 3. IL-1 RI, 4. NFκB2, 5. IL-17 RA, 6. TNF RI/TNFRSF1A, 7. RelA/p65, 8. FADD/MORT1, 9. IκBε, 10. LTBR/TNFRSF3, 11. SOCS6, 12. STAT1p91, and 13. IKK2/IKKβ. (**C**) Early MSCs were treated with IL-1α (10 ng/mL) or IL-8 (10 ng/mL) in a 10 cm dish for 72 h, followed by measuring protein levels of IKK2, phospho-serine 536 p65 subunit of NFκB (pS536-p65), p16 and p21 by Western blotting. Actin served as the protein loading control. Fold change to Actin with normalization is shown. Data are expressed as mean ± SD (*n* = 3). Asterisks indicate significant differences as determined by the unpaired Student’s *t*-test (* *p* < 0.05, ** *p* < 0.01, *** *p* < 0.001 and **** *p* < 0.0001 versus control group, CTR).

IL-1α/IL-1R or IL-8/IL-8R signaling pathways are mediated through NF-κB, a key transcription factor, which are involved in many aging-related diseases [32]. To study the relationship between IL-1α, IL-8, NF-κB and senescence markers, the proteome profile of the human NF-κB pathway was performed in IL-1α−, or IL-8-treated early MSCs (Figure 5A). The quantification of mean pixel intensity demonstrated the increase in NF-κB-activated molecules, such as IKK2, NF-κB1, NF-κB2, and RelA/p65, and the increase in NF-κB-related molecules, such as STAT2, IL-1RI, IL17RA, FADD/MORT1, STAT1p91, CD40/TNFRSF5 and IL-18 (Figure 5B).

Because IκB kinase (IKK) activates the phosphorylation of NF-κB at Ser536 (pS536) [33], protein levels of NF-κB-activated molecules were further confirmed, as well as phospho-NFκB, p16 and p21 in IL-1α, or IL-8-treated early MSCs by Western blot (Figure 5C). These data indicate that IL-1α, or IL-8-induced senescence pathway, may be through NF-κB activation.

### 3.5. Inhibition of NF-κB Eliminates Paracrine Effect of SASP-Related Proinflammatory Cytokines on Early MSCs

**Figure 6:**
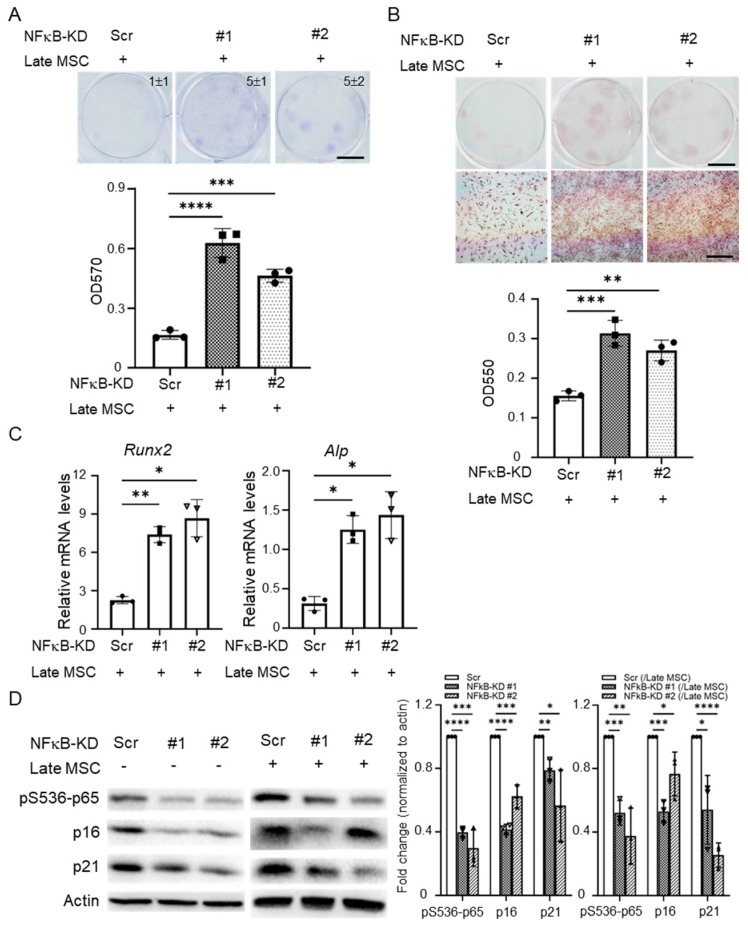
NFκB knockdown abolishes paracrine effect of late MSCs on senescence induction. Early MSCs were infected with lentivirus carrying scramble (Scr) or shRNAs against NFκB (NFκB-KD #1 or #2) plasmid, followed by puromycin selection for 2 days. (**A**) A total of 100 early MSCs without (Scr) or with NFκB-KD (#1 or #2) were seeded in each lower well of 6-well plates in the presence of 100 late MSCs seeded in the upper well of Transwell (0.4 μm pore size insert) and cultured for 14 days. The cells were stained with crystal violet (upper) and the dye was extracted for OD measurement at 570 nm (lower). Bar = 10 mm. (**B**) A total of 100 early MSCs without or with NFκB-KD were seeded in each lower well of 6-well plates in the presence of 100 late MSCs seeded in the upper well of Transwell and cultured for 7 days, and replaced with osteogenic induction media for an additional 14 days. The cells were stained with Alizarin Red-S (upper) and the colony number for each well is shown in right upper site. The dye was extracted for OD measurement at 550 nm (lower). Data are expressed as mean ± SD (*n* = 3). Bar = 10 mm (upper); 200 μm (lower). Asterisks indicate significant differences as determined by One Way ANOVA (* *p* < 0.05, ** *p* < 0.01, *** *p* < 0.001 and **** *p* < 0.0001 versus Scr). (**C**) A total of 5 × 10^4^ early MSCs without (Scr) or with NFκB-KD (#1 or #2) were seeded in each lower well of 6-well plates in the absence or presence of 5 × 10^4^ late MSCs seeded in the upper well of Transwell and cultured in osteogenic induction media for 3 days. qPCR analysis of transcript levels for genes associated with osteogenesis. Transcript levels were normalized based on GAPDH amplification. Alkaline-p (Alp). *n* = 3 independent experiments. (**D**) A total of 5 × 10^4^ early MSCs with or without NFκB-KD were cultured in each lower well of 6-well plates in the absence or presence of 5 × 10^4^ late MSCs seeded in the upper well of Transwell and cultured for 72 h, followed by measuring protein levels of phospho-serine 536 p65 subunit of NFκB (pS536-p65), p16 and p21 by Western blotting. Actin served as the protein loading control. Fold change to Actin with normalization is shown. Data are expressed as mean ± SD (*n* = 3). Asterisks indicate significant differences as determined by One Way ANOVA (* *p* < 0.05, ** *p* < 0.01, *** *p* < 0.001 and **** *p* < 0.0001 versus Scr).

Lentivirus-mediated knockdown was further used to directly target and investigate the roles of NF-κB in paracrine effects of late MSCs. Early MSCs transduced with NF-κB shRNAs (NFκB-KD) in the Transwell co-culture system of late MSCs rescued clonogenic capacity (NFκB-KD #1 and #2: 5 ± 1 and 5 ± 2 > scramble: 1 ± 1, *p* value: 0.0105 and 0.0105, Figure 6A), osteogenic gene expression and osteogenic potentials into osteoblasts (Figure 6B,C), and reduced the level of NF-κB (pS536) and senescence markers (p16 and P21) (Figure 6D), compared to cells transduced with scrambled shRNA. Similarly, NFκB-KD MSCs in direct co-culture of late MSCs rescued clonogenic capacity (NFκB-KD #1 and #2: 6 ± 1 and 4 ± 2 > scramble: 3 ± 2, Figure S3A), and osteogenic capacity (Figure S3B). Furthermore, inhibition of NF-κB by shRNA or inhibitor (JSH-23), also reduced the level of NF-κB (pS536) and senescence markers in IL-1α or IL-8-treated early MSCs (Figures S4 and S5). Together, the data suggest that NF-κB is essential to induce senescence from SASPs and inhibit early MSC properties.

## 4. Discussion

In the present study, we have identified the altered characteristics of late MSCs and demonstrated important insights into the in vitro MSC aging process. Key pro-inflammatory cytokines, IL-1α and IL-8, were first identified by GSEA analysis as being associated with SASP in late MSCs. The paracrine effect of late MSCs was demonstrated using the Transwell system, which increased the expression of cellular senescence markers such as p16 and p21, reduced clonogenic capacity, and inhibited osteogenic differentiation potential of early MSCs. Furthermore, treatment of early MSCs with IL-1α and IL-8 induced senescence phenotypes through activation of the NF-κB signaling pathway. Findings of the present study suggest that treatment of early MSCs by inhibiting NF-κB activation reduces the paracrine effects of late MSCs, which are mediated by secretion of IL-1α and IL-8. Modulation of NF-κB signaling serves as a therapeutic target to counteract age-related paracrine effects.

Numerous studies have shown that senescence is associated with increased production of pro-inflammatory cytokines, such as IL-1 and IL-8, by senescent cells, collectively referred to as pro-inflammatory-associated SASP [34]. Notably, it is now evident that senescence can be transmitted to normal cells by SASP in a paracrine or autocrine manner [35]. IL-1 levels are upregulated in senescent cells and IL-1 is also involved in SASP-induced paracrine senescence [10]. Similarly, IL-1α was upregulated in late MSCs and contributed to the paracrine role of late MSCs in inducing senescence in early MSCs. IL-1α is also reported to be an upstream regulator of the senescence-associated IL-6/IL-8 cytokine network [31]. However, our data showed that IL-8 was also upregulated in late MSCs compared to early MSCs and contributed to the paracrine role of late MSCs in inducing senescence in early MSCs. Therefore, we speculate that, like IL-1α, IL-8 may be independently involved in late MSCs-induced early MSCs cell senescence. Similarly, SASP-associated IL-8 secreted by aged MSCs induces premature senescence by limiting the proliferation capacity of neighboring young hematopoietic stem cells (HSPCs) [13].

Mechanistically, in the present study, the direct causal relationship of SASP-related IL-8 to early MSC senescence was determined by protein array and validated by Western blotting. NF-κB controls the transcription of key regulators of key SASP factors, such as the proinflammatory cytokines IL-1α, IL-6, and IL-8, which in turn positively regulates NF-κB activity in an autocrine feedback manner to enhance SASP signaling [36]. IL-1α binds its cell surface receptor, initiating a signaling cascade of NF-κB activation, which results in cellular senescence [37]. In the present study, the levels of phosphorylated NF-κB were also increased in early MSCs treated with IL-1αor IL-8 compared with untreated early MSCs. In addition, NF-κB knockdown in early MSCs blocked the paracrine senescence of late MSCs, compared with scrambled shRNAs transduced in early MSCs.

The senescent state is primarily characterized by durable cell cycle arrest [34,38]. Activation of cyclin-dependent kinase (CDK) inhibitors p16 and p21 is essential for senescence-associated growth arrest, which antagonizes CDK to block cell cycle progression [34,38]. During cellular senescence, the expression level of p16 and p21 was shown to be upregulated both in vitro and in vivo, elevating SA-β-gal expression [6]. We and others have also shown that inhibition of p16 or p21 can also reverse MSC senescence and restore their stemness [23,39]. Additionally, the present study showed that late MSCs secreted IL-1α and IL-8 to increase the expression of p16, p21 and SA-β-gal in early MSCs. However, we extended these findings and further showed that knockdown of NF-κB activation in early MSC significantly reduces IL-1α- or IL-8-induced expression of p16, p21 and SA-β-gal. Moreover, after co-culture of late MSCs, levels of p16, p21 and SA-β-gal were significantly reduced in early MSCs transduced with NF-κB shRNA compared with that transduced with scramble shRNA. Taken together, we suggest that the paracrine senescence of late MSCs in early MSCs is mediated through activation of the NF-κB/p16/p21 pathway.

One study demonstrated that, during expansion, human bone marrow-derived MSCs from 8 donors reduced proliferation capacity [40], was associated with ‘‘in vitro-aging,’’ exhibited morphological abnormalities, skewed differentiation potential, attenuated surface marker expression and, finally, proliferative arrest [41,42]. Interestingly, similarities are found between these in vitro aging phenotypes and those observed in biologically aged human MSCs from aged donors, or “in vivo-aging” phenotypes [43]. This suggests that similar molecular mechanisms may lead to a dramatic decline in key functional properties, such as bone-forming capacity (resulting in osteoporosis) and loss of anti-inflammatory properties, during aging in vitro and in vivo [44]. Osteoporosis is reported to be an age-related disease, the detailed mechanism of which is unknown [45]. Therefore, results of this study provide evidence supporting that the rejuvenation of MSCs in vitro through blocking the NF-κB pathway may be a potential therapeutic strategy for the treatment of osteoporosis. Obviously, this deserves further investigation.

In the present study, using GSEA, q-RT-PCR and ELISA, the acquisition of the SASP-related pro-inflammatory phenotype (e.g., IL-1α and IL-8) in late MSCs was demonstrated compared to early MSCs. However, a low concentration of IL-1α protein was observed in conditional medium of MSCs by ELISA, although the activated effect of low dose as well as ELISA results by Western blot had been confirmed (Figure S5). Two possible explanations may account for this finding: (1) spatial cytokine concentration gradients in response to cellular senescence of MSCs and (2) the synergistic action of pro-inflammatory proteins. Currie et al. [46] quantified the cytokine concentrations in vessel and muscle tissues at sites both near and distal to the injury, at several time points post-fracture. Clear evidence of spatial concentration gradients-dependent concentrations is found in both tissues sampled. Kaplan et al. [47] suggested that a reasonable definition of physiological concentrations for autocrine cytokines refers to levels that are obtained in vivo at sites relevant for biological activity. These sites may be at the surface of the secreting cell or even in an intracellular compartment [47]. Consequently, it is possible that the paracrine in MSCs contains spatial cytokine concentration gradients in response to cellular senescence. In addition, Paludan et al. [48] indicated that the low concentration of pro-inflammatory cytokines had a synergistic action in cellular and molecular aspects. Ortiz-Montero et al. [49] also revealed that purified IL-6 and IL-8 induce a cross-reinforced senescence in cancer cells. In the present study, we observed that the activation of NF-κB pathway and the induction of cellular senescence was also enhanced in early MSCs treated with IL-1α plus IL-8 compared with that in early MSCs treated with IL-1α or IL-8 alone (Figure S6). This observation may also explain why a low concentration of IL-1α protein in conditional medium of MSCs induces cellular senescence.

## 5. Conclusions

Late MSCs display features of senescence and, during aging, SASP-related proinflammatory cytokines activate a cellular senescence program in early MSCs that may ultimately impair their functionality. Paracrine SASP-related proinflammatory cytokines play pivotal roles in the MSC in vitro-aging process based on the clarified molecular mechanism of late MSC-induced paracrine SASP-mediated senescence of early MSCs. Targeting the related molecules of this mechanism allows us to rescue the MSC in vitro aging process. Clinically, novel technologies and strategies can be designed to optimize MSC-based therapies of ageing-associated diseases by targeting the related molecules.

## Figures and Tables

**Table 1:** GSEA identifies differently expressed SASP genes of late MSCs.

Gene	Function	Log2 Fold Change	FDR
CDKN2C	Cyclin Dependent Kinase Inhibitor 2C encoded by this gene is a cyclin-dependent kinase inhibitors.	−2.146323063	2.53 × 10^−10^
H2BC4	H2B Clustered Histone 4 play a central role in transcription regulation, DNA repair, DNA replication and chromosomal stability.	3.908697986	6.37 × 10^−9^
H2BC5	H2B Clustered Histone 5 play a central role in transcription regulation, DNA repair, DNA replication and chromosomal stability.	2.851917638	2.47 × 10^−7^
H2AC6	H2A Clustered Histone 6 play a central role in transcription regulation, DNA repair, DNA replication and chromosomal stability.	2.443859801	7.27 × 10^−7^
IL1A	Interleukin-1 alpha encoded by this gene is involved in various immune responses, inflammatory processes, and hematopoiesis. Produced by activated monocytes and \macrophagesas a proprotein, IL-1 stimulates thymocyte proliferation by inducing IL-2 release, B-cell maturation and proliferation, and fibroblast growth factor activity.	5.090993692	2.87 × 10^−6^
H2AZ1	H2A.Z Variant Histone 1 encodes a replication-independent member of the histone H2A family that is distinct from other members of the family. This particular histone is required for embryonic development and indicate that lack of functional histone H2A leads to embryonic lethality.	−1.102308719	7.83 × 10^−6^
H2BC12	H2B Clustered Histone 12 play a central role in transcription regulation, DNA repair, DNA replication and chromosomal stability.	2.395549293	2.20 × 10^−5^
H3-3B	H3.3 Histone B play a central role in transcription regulation, DNA repair, DNA replication and chromosomal stability.	−1.069780573	0.001343697
H2BC21	H2B Clustered Histone 21 play a central role in transcription regulation, DNA repair, DNA replication and chromosomal stability.	2.426780384	0.002802481
H4C8	H4 Clustered Histone 8 play a central role in transcription regulation, DNA repair, DNA replication and chromosomal stability.	2.98060193	0.002899564
MAPK3	Mitogen-Activated Protein Kinase 3 extracellular signal-regulated kinases are a group of mitogen-activated protein kinases (MAPK) that mediate intracellular signaling. which enable the propagation the MAPK/ERK signal to additional cytosolic and nuclear targets, thereby extending the specificity of the cascade.	0.841703804	0.006413522
CXCL8	C-X-C Motif Chemokine Ligand 8 is a member of the CXC chemokine family and is a major mediator of the inflammatory response. The encoded protein is commonly referred to as interleukin-8 (IL-8). These small basic heparin-binding proteins are proinflammatory and primarily mediate the activation and migration of neutrophils into tissue from peripheral blood	3.313472036	0.011501879
H3C4	H3 Clustered Histone 4 play a central role in transcription regulation, DNA repair, DNA replication and chromosomal stability.	6.03191529	0.032235243
JUN	Jun Proto-Oncogene, AP-1 Transcription Factor Subunit is a transcription factor that recognizes and binds to the enhancer heptamer motif 5′-TGA[CG]TCA-3′.	0.71322982	0.032952434
CDKN2A	Cyclin Dependent Kinase Inhibitor 2A produces 2 major proteins: p16(INK4), which is a cyclin-dependent kinase inhibitor, and p14(ARF), which binds the p53-stabilizing protein MDM2.	1.277496987	0.035892102
H2BC8	H2B Clustered Histone 8 play a central role in transcription regulation, DNA repair, DNA replication and chromosomal stability.	5.345592369	0.03752821

FDR ≦ 0.05.

## Data Availability

The datasets used and/or analyzed during the current study are available from the corresponding author on request.

## Supplementary Materials

The following supporting information can be downloaded at: https://www.mdpi.com/article/10.3390/cells11203324/s1, Figure S1: Morphology, senescence marker expression and cell cycle function in early and late MSCs, Figure S2: Senescence-associated β-galactosidase staining showing senescent cells in early MSCs treated with IL-1α or IL-8, Figure S3: NFκB knockdown abolishes the senescence-inducing effect of late MSCs, Figure S4: NFκB knockdown abolishes the senescence-induction effect of IL-1α- or IL-8 on early MSCs, Figure S5: Inhibition of NFκB abolishes senescence-induction effect of IL-1α- or IL-8 on early MSCs, Figure S6: IL-1α and IL-8 synergistically activate NFκB pathway to increase the expression of senescence markers in early MSCs.
