# Supplementary material for: Paracrine Senescence of Mesenchymal Stromal Cells Involves Inflammatory Cytokines and the NF-κB Pathway
Source: Cells. 2022 Oct 21;11(20):3324. doi: 10.3390/cells11203324 (PMC9600401; doi:10.3390/cells11203324)
Supplement: Supplementary file 1 [file cells-11-03324-s001.zip › cells-1950551-supplementary.pdf]

Supplementary Information

Inventory:
Table S1-S2
Figure S1-S6

Table S1. Donor information

Donor No.	Age (years)/ gender	Source of stem cells
1	27/Female	Bone marrow
2	28/Male	Bone marrow
3	41/Male	Bone marrow

Table S2. Primer sets for real-time PCR

Name	Primer sequence
Runx2	F: TTACCTACACCCCGCCAGTC
	R: TGCTGGTCTGGAAGGGTCC
Alp	F: TGAGCGACACGGACAAGA
	R: GGCCTGGTAGTTGTTGTGAG
GAPDH	F: CAAATTCCATGGCACCGTCAA
	R: AGGGATCTCGCTCCTGGAAG

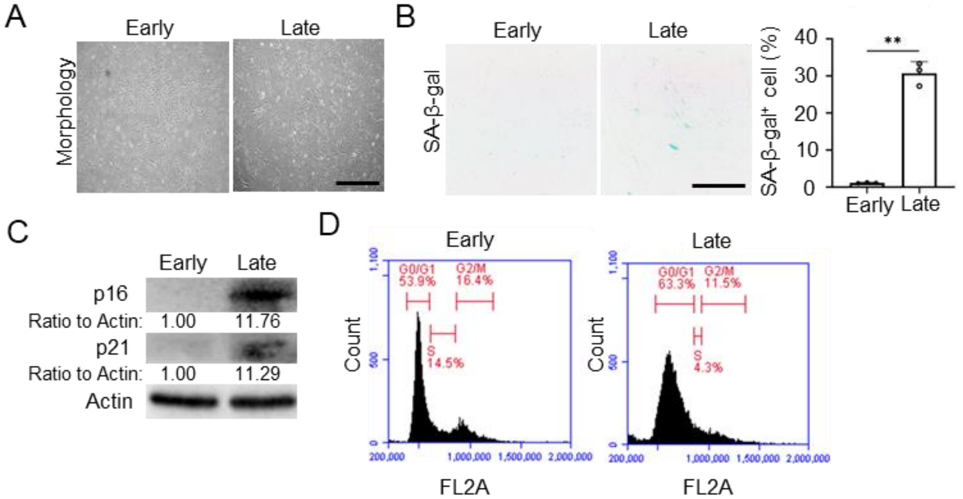

Figure S1. Morphology, senescence marker expression and cell cycle function in early and late MSCs. (A) Morphology of early-passage (P2-5), and late-passage (P9-14) MSCs. (B left) Senescence-associated β-galactosidase (SA-β-gal) staining showing senescent cells in late MSCs. Bar = 200 μm. (B Right) Quantification of SA-β-gal+ cell percentages. **p<0.01, as determined by the unpaired Student's t-test. Data are expressed as mean ± SD. (n = 3) (C) Westering blotting showing the increase of senescence markers, p16 and p21, in late MSCs. Ratio to Actin with normalization is shown. (D) Representative cell cycle distribution of early and late MSCs as analyzed by flow cytometry with PI staining. (n = 3)

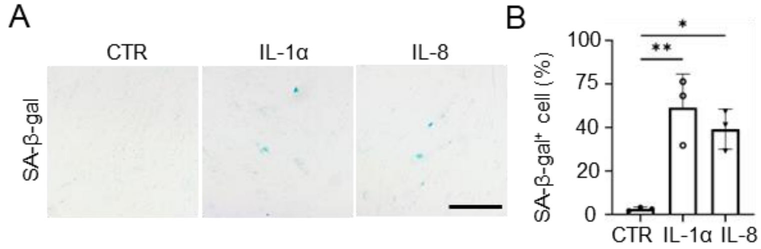

Figure S2. Senescence-associated β-galactosidase staining showing senescent cells in early MSCs treated with IL-1α or IL-8. (A) Early MSCs were treated with IL-1α (10 ng/mL) or IL-8 (10 ng/mL) and cultured for 72 hours, followed by Senescence-associated β-galactosidase (SA-β-gal) staining. Bar=200 µm. (B) Quantification of SA-β-gal+ cell percentages. Data are expressed as mean ± SD. (n = 3) *p<0.05 and **p<0.01, as determined by One Way ANOVA.

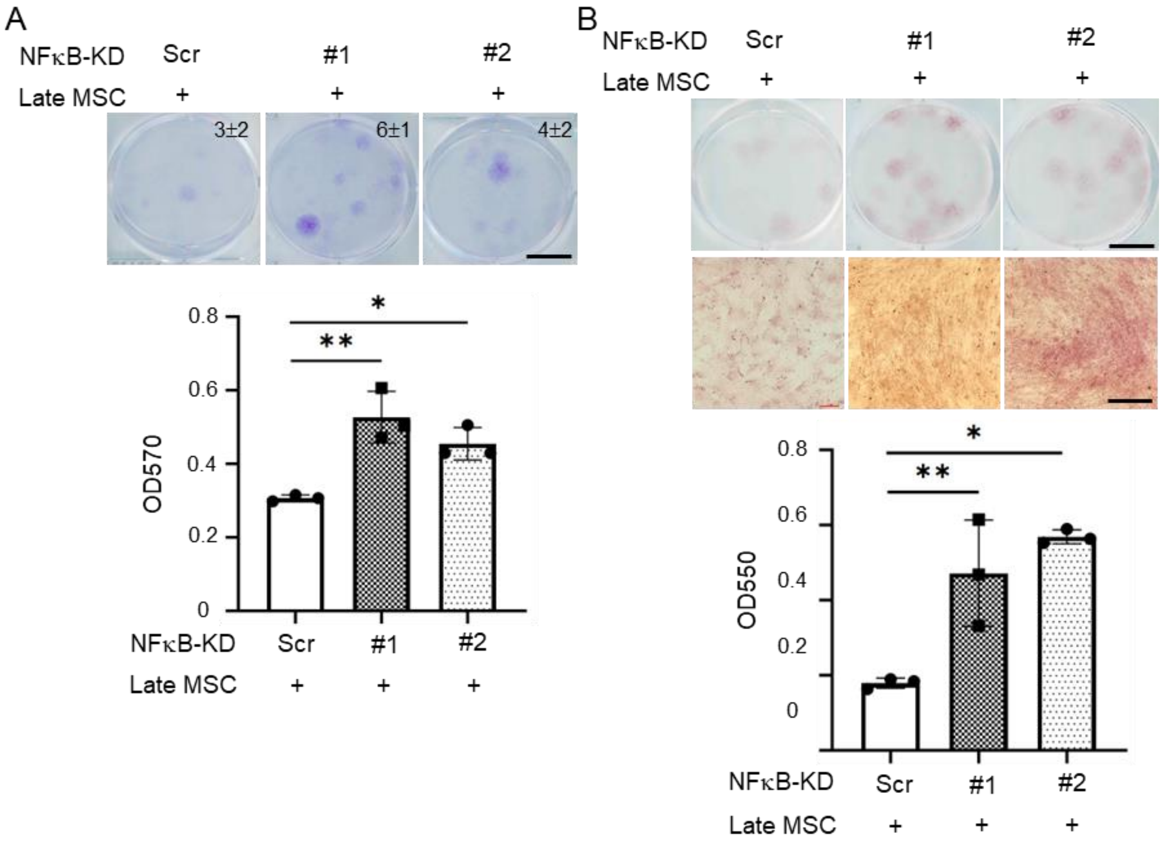

Figure S3. NFκB knockdown abolishes the senescence-inducing effect of late MSCs. Early MSCs were infected with lentivirus carrying scramble (Scr) or shRNAs against NFκB (NFκB-KD #1 or #2) plasmid, followed by puromycin selection for 2 days. (A) 100 early MSCs without (Scr) or with NFκB-KD (#1 or #2) were co-cultured with 100 late MSCs for 14 days. The cells were stained with crystal violet (upper) and the colony number for each well is shown in right upper site. The dye was extracted for OD measurement at 570 nm (lower). Bar = 10 mm. (B) 100 early MSCs without or with NFκB-KD were co-cultured with 100 late MSCs for 7 days, and replaced with osteogenic induction media for an additional 14 days. The cells were stained with Alizarin Red-S (upper) and the dye was extracted for OD measurement at 550 nm (lower). Bar = 10 mm (upper); 200 μm (lower). Data are expressed as mean ± SD (n = 3). *$p<0.05$ and **$p<0.01$, as determined by One Way ANOVA.

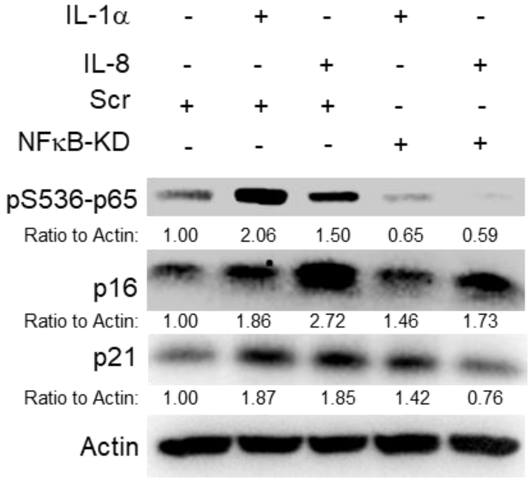

Figure S4. NFκB knockdown abolishes the senescence-induction effect of IL-1α- or IL-8 on early MSCs. (A) Early MSCs were infected with lentivirus carrying scramble (Scr) or shRNAs against NFκB (NFκB-KD #1 or #2) plasmid, followed by puromycin selection for 2 days. The cells were treated with IL-1α (10 ng/mL) or IL-8 (10 ng/mL) at 50% confluence for 72 hours, followed by measuring protein levels of phospho-NFκB, p16 and p21 by western blotting. Early MSCs at 50% confluence were treated JSH-23 (30 μM) with (A) IL-1α (10 ng/mL) or (B) IL-8 (10 ng/mL) in 10-cm dish for 3 days, followed by measuring phospho-serine 536 p65 subunit of NFκB (pS536-p65) and senescence markers, p16 and p21, by western blotting. Actin served as the protein loading control. Ratio to Actin with normalization is shown.

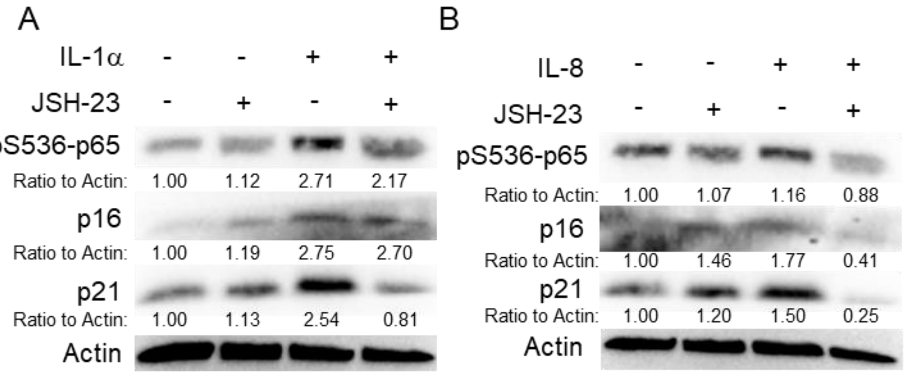

Figure S5. Inhibition of NFκB abolishes senescence-induction effect of IL-1α- or IL-8 on early MSCs. Early MSCs at 50% confluence were treated JSH-23 (30 μM) with (A) IL-1α (10 ng/mL) or (B) IL-8 (10 ng/mL) in 10-cm dish for 3 days, followed by measuring phospho-serine 536 p65 subunit of NFκB (pS536-p65) and senescence markers, p16 and p21, by western blotting. Actin served as the protein loading control. Ratio to Actin with normalization is shown.

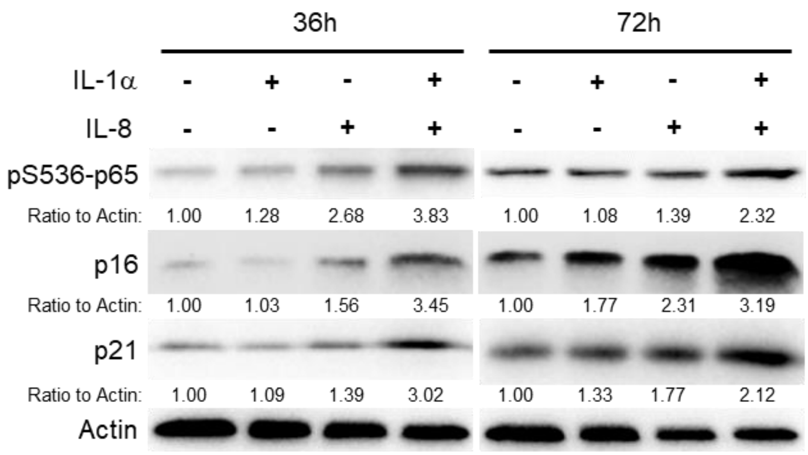

Figure S6. IL-1α and IL-8 synergistically activate NFκB pathway to increase the expression of senescence markers in early MSCs. Early MSCs were treated with or without IL-1α (0.001 pg/mL) or/and IL-8 (350 pg/mL) in 10-cm dish for 72 hours, followed by measuring protein levels of phospho-serine 536 p65 subunit of NFκB (pS536-p65), p16 and p21 by western blotting. Actin served as the protein loading control. Ratio to Actin with normalization is shown.
